# Sulforaphane for the chemoprevention of bladder cancer: molecular mechanism targeted approach

**DOI:** 10.18632/oncotarget.16015

**Published:** 2017-03-08

**Authors:** Andrew Leone, Gregory Diorio, Wade Sexton, Michael Schell, Mark Alexandrow, Jed W. Fahey, Nagi B. Kumar

**Affiliations:** ^1^ Genitourinary Oncology, H. Lee Moffitt Cancer Center & Research Institute, Inc., Tampa, FL, USA; ^2^ Biostatistics and Bioinformatics, H. Lee Moffitt Cancer Center & Research Institute, Inc., Tampa, FL, USA; ^3^ Cancer Biology and Evolution, H. Lee Moffitt Cancer Center & Research Institute, Inc., Tampa, FL, USA; ^4^ Clinical Pharmacology, Johns Hopkins School of Medicine, Baltimore, MD, USA; ^5^ Cancer Epidemiology, H. Lee Moffitt Cancer Center & Research Institute, Inc., Tampa, FL, USA

**Keywords:** sulforaphane, bladder cancer, chemoprevention, safety, toxicity

## Abstract

The clinical course for both early and late stage Bladder Cancer (BC) continues to be characterized by significant patient burden due to numerous occurrences and recurrences requiring frequent surveillance strategies, intravesical drug therapies, and even more aggressive treatments in patients with locally advanced or metastatic disease. For these reasons, BC is also the most expensive cancer to treat. Fortunately, BC offers an excellent platform for chemoprevention interventions with potential to optimize the systemic and local exposure of promising agents to the bladder mucosa. However, other than smoking cessation, there is a paucity of research that systematically examines agents for chemoprevention of bladder cancers. Adopting a systematic, molecular-mechanism based approach, the goal of this review is to summarize epidemiological, *in vitro*, and preclinical studies, including data regarding the safety, bioavailability, and efficacy of agents evaluated for bladder cancer chemoprevention. Based on the available studies, phytochemicals, specifically isothiocyanates such as sulforaphane, present in Brassicaceae or “cruciferous” vegetables in the precursor form of glucoraphanin are: (a) available in standardized formulations; (b) bioavailable- both systemically and in the bladder; (c) observed to be potent inhibitors of BC carcinogenesis through multiple mechanisms; and (d) without toxicities at these doses. Based on available evidence from epidemiological, *in vitro*, preclinical, and early phase trials, phytochemicals, specifically isothiocyanates (ITCs) such as sulforaphane (SFN) represent a promising potential chemopreventitive agent in bladder cancer.

## INTRODUCTION

Bladder cancer (BC) is the fourth most common cancer in men in the United States and eighth most common cause of cancer death [1]. In 2016, an estimated 76,000 men and women will be diagnosed with BC in the US and 16,000 people will die of BC [2]. Cigarette smoking (mainly exposure to aromatic amines) accounts for 50% of bladder cancers [3, 4]. Non-tobacco related occupational exposure to amines, 4-aminobiphenyl & anilines (10% of all cases), as well as phenacetin derived analgesics (oral pain medications) [5, 6] have also been known to contribute to the etiology of BC. BC originates primarily in the transitional cell epithelium (urothelial epithelium) that lines the inner surface of the bladder and is directly exposed to urine, which is also known as transitional cell carcinoma (TCC) [4]. Approximately 70-75% of newly diagnosed BCs are non-muscle invasive (NMIBC), previously referred to as “superficial” BC, while 25-30% of tumors upon initial diagnosis are muscle invasive (≥ clinical stage T2) [4]. NMIBC is typically treated with endoscopic transurethral resection (TUR), which may be followed by an intravesical therapy, depending on the extent of the cancer, tumor grade and the presence of carcinoma *in situ*. Given the high risk of recurrence and disease progression, careful surveillance after cancer removal by TUR *via* cystoscopy is currently the standard clinical practice. Intravesical therapies with Bacillus Calmette-Guerin (BCG) or chemotherapeutic agents (e.g., mitomycin C), delivered *via* a urethral catheter, are used to prevent or delay recurrence and progression after TUR [7]. Although BCG has been more effective than other agents, 20-40% of patients fail to respond [8]. Recurrence is common despite BCG treatment with recurrence rates for high risk T1 tumors ranging from 16 to 40% and progression rates of 4% to 40%. [9–14]. Upon diagnosis of muscle invasive bladder cancer (stage T2), the current definitive treatment is radical cystectomy (surgical extirpation of the bladder) and urinary diversion. Overall survival is poor once distant metastasis (~15% 5 year survival) has occurred [15] with stage being the most important prognostic factor of BC [16].

The clinical course for both early and late stage BC continues to be characterized by significant patient burden due to numerous occurrences and recurrences requiring frequent surveillance strategies, intravesical drug therapies, and even more aggressive treatments in patients with locally advanced or metastatic disease Additionally, BC is the most expensive overall cancer to treat given its propensity to recur and the need for frequent treatment and surveillance [17, 18]. BC thus carries a significant patient burden as well as a healthcare cost-related burden underscoring the need to optimize BC care and need for prevention strategies especially targeting non-muscle invasive patients [17, 18]. Evaluation of chemoprevention interventions in BC patients is especially feasible given physiological exposure of bladder urothelial cells to excreted compounds, readily available pathological specimens for analysis, and measurable intermediate endpoint biomarkers [17, 19]. However, other than smoking cessation, there is a paucity of research that systematically examines agents for the chemoprevention of BC [20]. Smoking cessation has been shown to decrease recurrence and improve prognosis, yet this beneficial effect is only observed for long term smoking cessation ( > 10 years) [17, 19].

The objective is to review the available evidence from epidemiological, *in vitro*, pre-clinical animal and early clinical trials of various agents evaluated for bladder cancer chemoprevention with a focus on sulforaphane for bladder cancer chemoprevention.

## RESULTS

### Current strategies for bladder cancer chemoprevention

### Vitamins

Previous studies have focused on vitamin intake including, vitamin C, vitamin B6 and vitamin E and an essential trace element, selenium. However, the majority of these studies failed to indicate a promising agent for primary or secondary chemoprevention of BC [21]. Retinoids (vitamin A derivatives) and alpha tocopherol (vitamin E) have been studied as a putative chemopreventive agent in bladder cancer. *In vitro* studies suggested that rats with vitamin A deficiency were more likely to develop environmentally induced bladder cancer, and that supplementation of vitamin A could prevent bladder cancer development [22–25]. However, clinical studies do not support a chemopreventive role of retinoids, including the ATBC study that targeted at-risk smokers and assigned patients to beta-carotene, alpha tocopherol, both or placebo and showed no benefit in prevention of bladder cancer at 6 years of follow up [26]. A secondary analysis of the SELECT trial also failed to show a protective effect for vitamin E or selenium for bladder cancer [27]. Other studies exploring the role of retinoids for secondary chemoprevention also showed no benefit, and as a result of concerns for toxicity (increased myocardial infarction risk) and lack of clear benefit, one study was terminated prior to accrual [28–30].

Pyridoxine (B6) has been investigated in two randomized trials for secondary chemoprevention without evidence of a benefit [31, 32]. Ascorbic acid (vitamin C) has not been studied in randomized trials, and epidemiological data is not convincing with respect to its protective effect [33]. Mega dose multivitamins have not demonstrated clinical effectiveness in chemoprevention despite epidemiological research and clinical research suggesting a possible role for chemoprevention [34].

### NSAIDS and Cox-2 inhibitors

More recent chemopreventive efforts have exposed the role of non-steroidal anti-inflammatory drugs, specifically the role of selective COX-2 inhibitors. This has included clinical studies with celecoxib that suggested a correlation between COX-2 expression and prognosis. One trial in nonmuscle invasive bladder cancer patients showed similar risk in progression and recurrence between celecoxib and placebo [35]. The results of an expanded Phase III clinical trial in non-muscle invasive bladder cancer patients who responded to BCG treated with celecoxib or placebo are still not available. Intriguingly, an *in vitro* study using allyl isothiocyanate (AITC) with celecoxib produced depletion of prostaglandin E2, a key downstream signaling molecule of Cox-2, caspase activation and down regulation of vascular endothelial growth factor in the tumor tissues. These data show that AITC and celecoxib may complement each other in inhibiting bladder carcinogenesis, providing a novel combination approach for future validation in preclinical models for chemoprevention of bladder cancer [36].

**Table 1:** Clinical trials assessing sulforaphanes for cancer chemoprevention

Agent	Dose/Duration	Cancer	Sample size	Endpoints
60 mg (340 μmol) “stabilized SFN” (Prostaphane®) vs. Placebo [73]	6 months (RCT)	Prostate (Rising PSA after prostatectomy)	N=81	Lower Log PSA slope in the SFN group (p = 0.01)and serum PSA (p=<0.05) compared to placebo
200 μmol daily [105]	5 months	Prostate (Rising PSA after prostatectomy)	N=20	1 of 20 patients with 50% decline in PSA at 5 months
400 g broccoli/week vs. 400 g peas/week [123]	12 months	Prostate (Patients with high-grade prostate intraepithelial neoplasia) (HGPIN),	N=22	Significant changes in TGFβ, Insulin signaling and EGF receptor pathways
Glucoraphanin (30 mg GFN BroccoMax™ vs. placebo)[76]	2-8 weeks	Breast (Abnormal mammograms/pre-biopsy)	N=27	Ki-67 (*p* = 0.003) and HDAC3 (*p* = 0.044) levels significantly decreased in benign tissue. GFN supplementation was associated with a significant decrease in PBMC HDAC activity (*p* = 0.04).

### EGFR inhibitors and mTOR inhibitors

Also, research is being conducted with erlotinib, highly selective, reversible inhibitor of epidermal growth factor receptor (HER1/EGFR) tyrosine kinase which is overexpressed in more than 75% of bladder cancers [37]. One phase 2 clinical trial involved neoadjuvant administration of erlotinib in patients before undergoing radical cystectomy with a complete response rate in twenty five percent of patients. There was substantial skin toxicity noted especially in patients who experienced complete response [38]. A phase IIa randomized multi-institutional trial (NCT02169284) is ongoing investigating role of erlotinib in presurgical (RC or TURBT) patients [39] .

Additionally, research has focused on mTOR inhibition as a potential target for chemoprevention in bladder cancer [40]. Metformin, a commonly utilized diabetes medication has properties as an mTOR inhibitor and has been investigated with underwhelming results mostly in a retrospective fashion [41, 42]. One nonrandomized clinical trial comparing non muscle invasive BC patients taking metformin to placebo showed no difference in recurrence and no statistical difference in time to recurrence [43]. Also, *in vitro* research using *Rhodiola rosea* extract has shown to inhibit mTOR and decrease growth of bladder cancer [42]. Finally, some research has demonstrated a possible role of estrogen receptor blockade using tamoxifen in mouse model in modulating bladder tumorigenesis [44].

### Soy isoflavones

Isoflavones, which are soy derivatives, have recently been investigated as a possible chemoprevention agent based on epidemiological and *in vitro* evidence. A recent trial by Messing et al., explored the use of genistein, an isoflavone, in pre-surgical bladder cancer patients and demonstrated significant inhibition of p-EGFR at dose-specific levels, but other apoptotic and proliferative biomarkers were not impacted [45]. There has been an increase in newer research that has focused on isolation of (natural) bioactive compounds. Recently, Justicidin A, a methanol extract of *Justicia procumbens*, has been investigated *in vitro* as an anti-angiogenic and apoptosis-inducing agent [46], as have pomegranate extracts [47]. Additionally, green tea catechins have been demonstrated in several *in vitro* and *in vivo* studies to have significant anti-carcinogenic potential [48–51]. However, these are early observations that have to be further validated in preclinical models prior to evaluation in clinical trials.

Despite attempts at identifying other single and combination agents for chemoprevention, several epidemiological, *in vitro*, preclinical, and early phase trials have shown that the phytochemicals, isothiocyanates (ITCs), specifically sulforaphane (SFN) present in Brassicaceae or “cruciferous” vegetables in the precursor form of glucoraphanin [52–54], are: (a) available in standardized formulations; (b) bioavailable - both systemically and in the bladder; (c) observed to be potent inhibitors of BC carcinogenesis through multiple mechanisms [2]; and (d) associated with no dose-limiting toxicities at the proposed dose levels, thus supporting further development of SFN in phase I/II human studies targeting bladder cancer.

### Sulforaphane for bladder cancer chemoprevention

Sulforaphane (SFN), (−)-1-isothiocyanato-(4R)-(methylsulfinyl) butane [CH3-SO-(CH2)4-NCS], is an isothiocyanate found in high concentrations in broccoli sprouts. Sulforaphane was first isolated and shown to be a potent anti-carcinogenic agent in 1992 by Zhang, et al. [54].

### Epidemiological evidence for sulforaphane for bladder cancer chemoprevention

Epidemiological studies have shown a potential role for increased fluid intake and consumption of cruciferous vegetables, particularly for broccoli consumption, in reducing the risk of BC [55–57]. In a large prospective study, 39% reduction in BC risk was observed with an intake of 2 servings or more of broccoli compared to < 1 serving per week (*p* = 0.0009) [55]. In a meta-analysis of ten clinical trials, cruciferous vegetable intake was associated with decreased risk of bladder cancer overall [58].

### Pharmacokinetics of sulforaphane

Given the epidemiological studies which suggested a potential role for SFN as a chemoprevention agent, many studies have been conducted to elicit the pharmacokinetics of SFN. Broccoli accumulates significant amounts of the phytonutrient glucoraphanin (4-methylsulfinylbutyl glucosinolates), which is metabolized *in vivo* to the biologically active sulforaphane. This conversion requires myrosinase, which is present in the plant as well as in the gastrointestinal tract [53]. Upon being consumed, SFN is metabolized *via* the mercapturic acid pathway to form cysteinylglycine-, cysteine-, and N-acetylcysteine (NAC) conjugates. These metabolites are then excreted *via* the urine [59, 60]. Studies have shown that 70% of an initial SFN dose was able to be retrieved in urine [61, 62]. Urine has been shown in rat models to have significant concentration of SFN present as NAC conjugates with 72% to 95% of the original SFN dose recovered in urine [63] Also, bladder tissue in a rat model has been shown to have very high concentrations of SFN after gastric lavage, second in visceral organ concentration only to stomach tissue [64]. Uptake of SFN into bladder cancer cells is dependent upon diffusion and rapidly conjugate with GSH and other intracellular proteins [65]. Several factors including concentration, lipophilicity, and exposure time influence uptake [65–67].

Preclinical studies using animal models have demonstrated bioavailability of SFN with metabolites distributed to all tissues, including the bladder, suggesting the potential for systemic benefits [68–70]. Administration of a freeze-dried aqueous extract of broccoli sprouts to rats significantly and dose-dependently inhibited bladder cancer development induced by N-butyl-N-(4-hydroxybutyl) nitrosamine [71]. The incidence, multiplicity, size, and progression of BC were all inhibited by the extract, while the extract itself caused no histologic changes in the bladder. Moreover, inhibition of bladder carcinogenesis by the extract was associated with significant induction of phase-II enzymes such as glutathione S-transferase and NAD(P)H:quinone oxidoreductase 1 in the bladder. Over 70% of the isothiocyanates present in the extract were excreted in the urine as isothiocyanate equivalents (isothiocyanates + dithiocarbamates) within 12 h after a single oral dose, indicating high bioavailability and rapid urinary excretion. Urinary concentrations in extract-treated rats were 2 to 3 orders of magnitude higher than those in plasma, indicating that the bladder epithelium, the major site of bladder cancer development, is most exposed to orally-dosed isothiocyanate. In a murine UMUC3 xenograft model, semi-purified diets containing 4% broccoli sprouts, or 2% broccoli sprout isothiocyanate extract, or gavaged pure SFN, or erucin (each at 295 μmol/kg, similar to dietary exposure) produced tumor weight reduction of 42% (*p* = 0.02), 42% (*p* = 0.04), 33% (*p* = 0.04), and 58% (*p* < 0.0001), respectively. SFN and erucin metabolites are present in mouse plasma (micromolar range) and tumor tissue, with N-acetylcysteine conjugates as the most abundant [68].

Several clinical trials have been conducted to evaluate the effectiveness of SFN for chemoprevention, most of which have investigated bioavailability in healthy, disease-free subjects [72–75,125]. The method of ingestion in these clinical trials has varied from pure SFN, broccoli soups/pill forms, and broccoli as a food item. Glucoraphanin (GRR) in broccoli is converted to SFN either by plant myrosinases, or if the plant myrosinases have been denatured by cooking, by bacterial myrosinases in the human colon. SFN is passively absorbed and rapidly conjugated with glutathione by glutathione S-transferases (GSTs), then metabolized sequentially by γ-glutamyl-transpeptidase (GTP), cysteinyl-glycinease (GCase) and N-acetyltransferase (NAT). The conjugates are actively transported into the systemic circulation where the mercapturic acid and its precursors are urinary excretion products. Deconjugation may also occur to yield the parent isothiocyanate, SFN. The mercapturic acid and cysteine conjugate forms are the major urinary metabolites of SFN [76]. In interventions with glucosinolate-containing Brussels sprouts for 1-3 weeks, increased GST enzyme activity with increased GST-alpha induction was observed in plasma and tissues including liver, bladder, and small intestine [72, 77]. Bioavailability, as measured by urinary excretion of SFN and its metabolites (in approximately 12-hour collections after dosing), was substantially greater with the SFN-rich (mean = 70%) than with GRR-rich (mean = 5%) beverages. Inter-individual variability in excretion was considerably lower with SFR than with GRR beverage [62]. These studies have also corroborated the critical role of myrosinase in metabolizing SFN, as patients taking food sources *vs*. extract of SFN without myrosinase had a fourfold increase in urinary concentration [76].

### Pharmacodynamics/ mechanisms of action of sulforaphane

**Figure 1:**
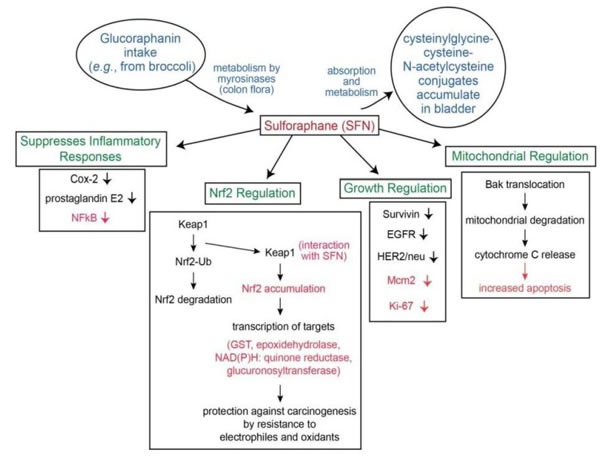
Metabolism and mechanism of SFN for bladder cancer chemoprevention

Although several molecular targets in cellular and animal models have been identified, the most sensitive target for SFN is Keap1, a key sensor for the adaptive stress response system regulated through the transcription factor Nrf2 (nuclear factor (erythroid-derived2)-like 2). Keap1 is a sulfhydryl-rich protein that represses Nrf2 signaling by facilitating the polyubiquitination of Nrf2, thereby enabling its subsequent proteasomal degradation. Interaction of SFN with Keap1 disrupts this function and allows for nuclear accumulation of Nrf2 and activation of its transcriptional program (Figure 1). Enhanced transcription of Nrf2 target genes provokes a strong cytoprotective response that enhances resistance to carcinogenesis and other diseases mediated by exposures to electrophiles and oxidants [78, 79,109]. Inhibition of Phase I enzymes by SFN may contribute to inhibition of procarcinogens and its chemopreventitive effect. The Nrf2 transcription factor is essential for induction of phase 2 proteins [78, 79, 109]. In *in vitro* and *in vivo* using murine models in oral carcinogenesis, SFN was demonstrated to activate the NRF2 pathway and downregulate oxidative damage [110–11]. SFN has also been observed to be a potent inducer of phase 2 detoxification enzymes [e.g., glutathione transferases, epoxide hydrolase, NAD(P)H: quinone reductase, and glucuronosyltransferases], which may offer protection against carcinogenesis, mutagenesis, and other forms of toxicity elicited by electrophiles and reactive forms of oxygen [69, 79–82] . SFN promotes reactive oxygen species formation by inhibition of Complex III within mitochondria [112] . Evidence suggests that SFN activation of production of 4-hydroxynonenal (a lipid peroxidation product) may be essential in the chemopreventitive mechanism. 4-hydroxynonenal induces proteins critical to apoptosis, promotes cell cycle arrest, and also activates Nrf2 pathway [113]. Other mechanisms include down regulation of NFkB, resulting in induction of cell cycle arrest and apoptosis [83], while selectively targeting abnormal/malignant cells [70] compared to normal BC cells [2, 36] . Using bladder cancer cell lines, Abbaoui, et al. [68] demonstrated that downregulation of survivin, epidermal growth factor receptor (EGFR), and human epidermal growth factor receptor 2 (HER2/neu) induced G2/M cell cycle accumulation and apoptosis [68]. In osteosarcoma cell lines, SFN decreased cell invasion and also focal adhesion kinase (FAK), both of which are important in cancer progression [114]. SFN has been shown to inhibit inflammatory responses, including downregulation of cyclooxygenase-2 (Cox-2) and reduction of prostaglandin E2 levels [84, 85]. SFN likely effects immune and inflammatory response specifically *via* Toll-like receptor (TLR4) suppression *via* blockade of thiol-mediated conjugation in macrophages [86]. Telomere length which is essential for cell survival may be decreased by exposure to SFN [115].

Other studies suggest a key mechanism involving SFN is disruption of mitochondria. SFN has been shown to promote translocation of Bak, leading to loss of transmembrane potential, mitochondrial degradation, and release of cytochrome C [78, 87]. Studies using bladder cancer cell lines suggest an M phase arrest as the primary mechanism for SFN [88]. In cervical cancer cell lines, SFN was shown to cause G2/M arrest *via* cyclinB1 downregulation [116]. Therefore, SFN acts upon a multitude of growth-regulatory and inflammatory pathways, some of which contain oncogenic targets, to exert its potent anti-cancer effects.

### *In vitro* models in bladder cancer

*In vitro* studies in BC [2, 89, 90], lung [91, 121], prostate [92, 93], colorectal, thyroid [124], renal [113], ovarian [118] and leukemia cells lines [94, 95] have shown SFN to be a potent inhibitor of carcinogenesis through several molecular mechanisms [79], including in BC as discussed below. SFN inhibits the survival and proliferation of a wide array of animal and human BC cell lines [2, 96–98]. Also, SFN has been shown to be selectively more toxic to malignant urothelial cells (human) than normal urothelium [68]. Using BIU87 bladder cell lines Dang et al demonstrated that SFN downregulated NF-KB levels and upregulated Insulin-like growth factor-binding protein-3 (IGFBP-3) resulting in more apoptosis [99]. Islam et al, studied combination of SFN with acetazolamide in bladder cancer *in vitro* cell lines and xenografts. The authors showed diminished Ki-67, PHH3, and cyclin D1 and increases in cell cycle inhibitors p21 and p27 as well as decreases in Akt kinase activity [117]. Additionally, the studies noted downregulation of key metastatic proteins such as E-cadherin, N-cadherin, and vimentin.

### Animal models

Other preclinical trials have demonstrated positive results using murine models with SFN for breast [100], skin [101], prostate [92], oral [110], and pancreatic [70] cancers. Three studies have been conduct in mice and rats investigating the effect of SFN using a BC model. Abbaoui et al, using a mouse xenograft model showed G2/M phase arrest in models treated SFN [68]. Wang et al using the same murine xenograft documented tumor growth inhibition by 63% as well as a reduction in angiogenesis and an increase in immunological cellular response [103]. Using a rat orthotopic model, Munday et al demonstrated reduced incidence of BC by 61% and a decrease in tumor size with a delay in time to tumor progression [71].

Animal studies have suggested potential toxicity of isothiocyanates, including bladder hyperplasia [104]. However, this effect was likely secondary to overdosing. In fact, SFN have never been implicated as toxic to the bladder even in animal models using doses significantly higher than those used in clinical trials.

### Clinical trials in other cancer models

To date, data from early clinical trials with SFN have focused on prostate cancer chemoprevention [73, 105, 123] with the exception of a single trial in women with abnormal mammograms, using a pre-biopsy window of opportunity for intervention with SFN [120]. The doses and formulations of SFN and duration of intervention, target populations and biomarkers/endpoints selected in these trials vary significantly. In one study, 60 mg (340 μmol) “stabilized SFN” (Prostaphane^®^) *vs*. placebo [73] was used targeting men with biochemical recurrence of prostate cancer. A reduction in serum PSA was observed in 8/20 (40%) of prostate cancer patients with no clinical toxicities, compared to placebo [105].

Targeting a similar population with 200 μmol daily for 5 months, Alumkal et al [105]demonstrated that 1 of 20 patients had a 50% decline in serum PSA at 5 months.

Using 400 g broccoli/week *vs*. 400 g peas/week targeting men with high-grade prostate intraepithelial neoplasia) (HGPIN), Traka et al [123] showed significant changes in TGFβ, Insulin signaling and EGF receptor pathways.

A recent double blinded randomized trial in women scheduled for breast cancer patients compared glucoraphanin supplement (Glucoraphanin (30 mg GFN BroccoMax™ *vs*. placebo) [76] prior to women undergoing breast biopsy. The trial showed a significant difference in several relevant biomarkers including Ki-67 and HDAC3 [120]. To our knowledge, there are no clinical trials that have been reported using SFN for BC chemoprevention.

## DISCUSSION

There are some limitations that are inherent to using agents that target multiple mechanistic pathways that contribute chemoprevention effects. A pragmatic future direction to chemoprevention is to utilize a broad spectrum approach [106] that involves using bioactive formulations of botanicals (as single agents or in combination with other botanicals or biologics) that have been shown to produce robust targeting of relevant and multiple, but well characterized molecular pathways, without clinically limiting toxicity - an approach that may be more effective than agents evaluated to date. SFN, similar to other botanicals, target multiple signal transduction pathways that make it challenging to determine exactly the interplay of these mechanisms in chemoprevention. Future studies should continue to examine specifc mechanisms as well as the interplay of these mechanisms involved in modulating bladder carcinogenesis. Further characterization of the chemopreventive properties of sulforaphane is critical to our understanding these mechanisms that may identify targeted pathways of sulforaphane in bladder carcinogenesis. In addition, these findings may identify and inform selection of biomarkers in evaluating efficacy of sulforaphane in modulating bladder carcinogenesis.

Additionally, bladder cancer represents a very genetically heterogeneous cancer with more than fifteen oncogenes identified and more than 20 tumor suppressor genes with varying frequencies [107], As such, chemopreventive agents that are effective in some types of bladder cancers, may not be as effective in others. It is also recognized that these agents may have specific effects at different stages of tumor progression. This certainly seems to be true of NRF2 activation in many types of cancer including lung cancer, where advanced disease is actually associated with the accumulation of genetic alterations in KEAP1 and NF2EL2 that promote constitutive activation. (TCGA also identified similar NF2EL2 mutations in muscle-invasive bladder cancers) [121]. Thus, data obtained from any agent, including SFN, which is evaluated for a specific stage of tumor, may not be generalizable for all stages of tumor [108]. Future studies should include early phase trials including heterogenous patient populations, including non-muscle invasive and muscle-invasive BC patients, potentially block randomized to examine effectiveness in various stages of bladder tumorogenesis, Additionally, the effectiveness as well as the biological effects of these early trials should include bladder tumor cells as well as the normal appearing urothelium adjacent to bladder neoplasia since these data may inform strategies for preventing the field cancerization effect seen in progressive, high grade superficial bladder cancer. Thus, by using a well rationalized, systematic approach, rigorous experimental design that addresses challenges and limitations are critical to evaluating agents for cancer chemoprevention in early phase trials, prior to advancing to phase III trial.

To date, data from early clinical trials with SFN have focused on prostate cancer chemoprevention [73, 105, 123] with the exception of a single trial in women with abnormal mammograms, using a pre-biopsy window of opportunity for intervention with SFN [120]. The doses and formulations of SFN and duration of intervention, target populations and biomarkers/endpoints selected in these trials vary significantly. Other than smoking cessation, there is a paucity of research that systematically examines specific agents relevant for chemoprevention of bladder cancers. Despite extensive knowledge of potential targets of SFN (Nrf2 induction and downregulation of NF-kB), and resulting modulation of intermediate endpoint biomarkers (apoptosis and proliferation) relevant to bladder carcinogenesis, these agents have not been evaluated in clinical trials for bladder cancer chemoprevention. Future clinical trials should be informed from the promising pharmacokinetics and pharmacodynamics evidence that exists from current *in vitro* and preclinical studies to inform design of early phase chemoprevention trials target men and women with non-muscle invasive as well as invasive bladder cancers.

The clinical course for both early and late stage BC continues to be characterized by significant patient burden due to numerous occurrences and recurrences requiring frequent surveillance strategies, intravesical drug therapies, and even more aggressive treatments in patients with locally advanced or metastatic disease. Based on available evidence from epidemiological, *in vitro*, preclinical, and early phase trials, phytochemicals, specifically isothiocyanates (ITCs) such as sulforaphane (SFN) represent a promising potential chemopreventative agent in bladder cancer. These studies will ultimately inform development of chemoprevention interventions in both early (non-muscle invasive) and late invasive stage BC patients.

## MATERIALS AND METHODS

MEDLINE (Ovid), EMBASE (Ovid), AMED (Ovid), CINAHL (EBSCO) and the Cochrane Library databases were searched for epidemiological studies, *in vitro*, *in vivo* and phase I-II clinical trials on the topic of bladder cancer chemoprevention with a focus on sulforaphane in bladder cancer chemoprevention. Only clinical trials investigating sulforaphane for cancer chemoprevention that were completed and results published in the identified databases were located.
